# Mortality of young offenders: a national register-based follow-up study of 15- to 19-year-old Finnish delinquents referred for forensic psychiatric examination between 1980 and 2010

**DOI:** 10.1186/s13034-017-0174-3

**Published:** 2017-08-08

**Authors:** Nina Lindberg, Jouko Miettunen, Anni Heiskala, Riittakerttu Kaltiala-Heino

**Affiliations:** 1 Forensic Psychiatry, Helsinki University and Helsinki University Hospital, PO Box 590, 00029 HUS Helsinki, Finland; 2 Center for Life Course Health Research, University of Oulu, Oulu, Finland; 3 Medical Research Center Oulu, Oulu University Hospital and University of Oulu, Oulu, Finland; 4 School of Medicine, Tampere University, Tampere, Finland; 5 Department of Adolescent Psychiatry, Tampere University Hospital, Tampere, Finland; 6 Vanha Vaasa Hospital, Vaasa, Finland

**Keywords:** Death, Delinquency, Mortality, Offending, Psychopathology

## Abstract

**Background:**

The mortality rate of young offenders is high. Furthermore, mortality in young offenders is associated with psychiatric and substance use disorders. The primary aim of this national register-based follow-up study was to investigate the mortality rate of Finnish delinquents who underwent a forensic psychiatric examination between 1980 and 2010. As delinquency is not a solid entity, we further aimed to compare the risk of premature death among different subgroups of the delinquents; violent versus non-violent offenders, offenders with alcohol use disorders versus those with no such diagnoses, offenders with schizophrenia spectrum disorders versus conduct- and personality-disordered offenders, under-aged versus young adult offenders, and, finally, boys versus girls.

**Methods:**

We collected the forensic psychiatric examination reports of all 15- to 19-year-old offenders who were born in Finland and had undergone the examination between 1.1.1980 and 31.12.2010 (n = 606) from the archives of the National Institute of Health and Welfare and retrospectively reviewed them. For each delinquent, four age-, gender- and place of birth-matched controls were randomly selected from the Central Population Register (n = 2424). The delinquents and their controls were followed until the end of 2015. The median follow-up time was 23.9 years (interquartile range 15.3–29.5). We obtained the mortality data from the causes of death register. Deaths attributable to a disease or an occupational disease were considered natural, and those attributable to an accident, suicide or homicide were considered unnatural.

**Results:**

By the end of the follow-up period, 22.1% (n = 134) of the delinquents and 3.4% (n = 82) of their controls had died (OR 8.11, 95% CI 6.05–10.86, p < 0.001). Among boys, 22.0% (n = 121) of the delinquents and 3.7% (n = 81) of the controls had died (OR 7.38, 95% CI 5.46–9.95, p < 0.001). Male delinquents’ risk of unnatural death was almost 11-fold, of natural death more than twofold, and of unclear death more than fourfold compared to that of their controls. No girls had natural or unclear deaths, but 23.6% (n = 13) of the delinquents and 0.5% (n = 1) of the controls had died due to unnatural causes (OR 67.79, 95% CI 8.63–532.00, p < 0.001). The violent delinquents’ risk of premature death was twice that of the non-violent delinquents. The other comparisons demonstrated no statistically significant differences between subgroups.

**Conclusions:**

Even though the Finnish correction system prefers psychiatric treatment and rehabilitation over criminal sanctions, and the national health care system offers developmental-phase-specific psychiatric care, the mortality rate of delinquents, especially of those with a history of violent offences, is high. The excess mortality of offenders can be regarded as a specific public-health inequity that calls for more effective intervention procedures than those used thus far.

## Background

The premature mortality of young offenders is high. For example, a follow-up study by Coffey et al. [1] reported an overall standardized mortality ratio (SMR) of 9.4 [95% confidence interval (CI) 7.4–11.9] for male and 41.3 (95% CI 20.2–84.7) for female delinquents aged 10–20. The link between criminal behavior and excess premature mortality remains throughout life: a meta-analysis by Zlodre and Fazel [2] showed SMR ranges between 1.0 and 9.4 for men and between 2.6 and 41.3 for women following release from prison. Premature death is often attributable to accidents, suicides, homicides [3–5], infections [4], and substance abuse [4], and is commonly associated with violence [3–5]. According to a 50-year follow-up study by Laub and Vaillant [4], along with aging, the aforementioned causes of death are accompanied by medical conditions and diseases such as cancers and cardiovascular diseases. The mortality rate varies depending on the severity of justice system involvement: arrested youth has the lowest rate of mortality, followed by detained and incarcerated youth [6]. Moreover, high delinquency severity shows earlier and higher mortality risk [7].

Juvenile delinquency is strongly linked to psychiatric morbidity. Between about half and two thirds of young offenders meet the diagnostic criteria for a conduct disorder, and between about a third and a half for a substance-use disorder [8–10]. Moreover, both borderline [11, 12] and antisocial [13] personality disorders associate highly with criminality. Findings on the prevalence of psychoses seem contradictory, but according to a meta-analysis of 25 surveys focusing on detained adolescents, it seems to be about three percent [10]. People with mental health disorders show excessive mortality [14]. Severe conduct-disordered behavior is associated with an increased risk of premature death, mostly due to accidents, suicide and homicide [15]. Recently, a community-based follow-up study highlighted the association between adolescent conduct problems and premature death attributable to cardiovascular diseases and cancers [16]. Both borderline and antisocial personality disorders relate strongly to impulsive and self-disruptive behavior [17–20], leading to an elevated risk of unnatural death. Antisocial personality disorder is often linked to Cloninger’s type 2 alcoholism [21] leading to high mortality from alcohol-related disorders [22]. According to a study by Hoye et al. [23], men with personality disorders also suffer higher mortality due to somatic disorders other than those related to alcohol than the general population, most probably due to underreporting of somatic symptoms and less help-seeking. With regard to people with schizophrenia spectrum disorders, cohort studies have revealed that the mortality rate for diseases and medical conditions is more than double than that of the general population [24, 25]. Among delinquents, hospitalization for a psychiatric disorder or substance abuse associates significantly with the risk of premature death [3].

The number of juvenile prisoners in Finland has traditionally been low by international standards because of the widespread use of conditional prison sentences and special youth punishments, including social rehabilitation and occupational activities [26]. Under-aged delinquents with severe offending are typically referred to child-welfare institutions, which focus on rehabilitation rather than correction. For example, there were only 11 under-aged individuals in Finnish prisons in 2015, (Criminal Sanctions Agency, personal communication). Further, according to Finnish law, courts can send an individual for a pretrial forensic psychiatric examination to determine whether he/she is suffering from a mental disorder that might affect his/her criminal responsibility. The final forensic psychiatric report includes an opinion on the offender’s level of criminal responsibility, as well as possible psychiatric diagnoses and an assessment as to whether or not the offender fulfils the criteria for involuntary psychiatric care. For example, offenders with schizophrenia spectrum disorders are typically referred for involuntary psychiatric treatment. Finland was the first country in Europe to acknowledge adolescent psychiatry as an independent specialty in the field of medicine with its own service regime: It was a distinct subspecialty in psychiatry between 1979 and 1994, until it became an independent medical specialty. Since then, both education and research on adolescent mental health disorders and their treatment have gained strength in Finland.

In this national register-based follow-up study, we collected a consecutive sample of Finnish delinquents who were sent for a pretrial forensic psychiatric examination between 1980 and 2010, and followed them until the end of 2015. Our primary aim was to investigate their mortality rate. We assumed that due to the above-mentioned special features related to Finnish judicial practices and mental health system, we would observe lower mortality rates than those reported by Coffey et al. [1]. Secondly, as delinquency is not a solid entity, we wanted to compare the risk of premature death among different subgroups of delinquents; violent versus non-violent offenders, offenders with alcohol use disorders versus those with no such diagnoses, offenders with schizophrenia spectrum disorders versus conduct- and personality-disordered offenders, under-aged versus young adult offenders, and finally, boys versus girls. On the basis of earlier research findings, we hypothesized that delinquents with violent offending would show significantly higher premature mortality than those with non-violent offending, and that delinquents with early onset alcohol abuse/dependence would have a significantly higher premature mortality rate than those without these disorders. We also assumed that, as delinquents with schizophrenia spectrum disorders are referred to involuntary psychiatric inpatient care, their mortality rate would be significantly lower than that of delinquents with conduct and personality disorders. We further assumed that, as under-aged delinquents with severe offending are referred to child-welfare institutions, their mortality rate would be significantly lower than that of their adult counterparts. The premature mortality rate of Finnish males is known to be approximately twice that of females [27]. We hypothesized that we would observe the same kind of gender difference in our offender population.

## Sample and methods

### Sample and procedure

The minimum age of criminal liability in Finland is 15 years. The National Institute of Health and Welfare organizes forensic psychiatric examinations. These examinations are inpatient evaluations that last approximately 2 months, and include data gathered from various sources (the juvenile him/herself, family members, relatives; and medical, criminal, school, child-welfare and military records), psychiatric evaluations, standardized psychological tests, interviews conducted by a multi-professional team, an evaluation of the offender’s physical condition, and continuous observation by the hospital staff. The overall high quality and reliability of Finnish forensic psychiatric examinations are acknowledged in the courts and among scientists [28]. The International Classification of Diseases—Eighth revision (ICD-8) [29] was used for the purposes of psychiatric classification in Finnish clinical practice between 1968 and 1986. It was replaced by the Diagnostic and Statistical Manual of Mental Disorders—Third revised edition (DSM-III-R) [30] which was used between 1987 and 1995. However, the diagnoses were converted into ICD-9 [31] diagnoses, e.g. when reporting them to the care register for health care. The ICD-10 [32] has been used since 1996.

We collected psychiatric examination reports on all 15- to 19-year-old offenders who were born in Finland and underwent the examination between 1.1.1980 and 31.12.2010 from the archives of the National Institute of Health and Welfare. The Ethics Committee of Helsinki University Hospital and the pertinent institutional authorities approved the study protocol.

### Participants

The study participants comprised 606 delinquents (girls: 9.1%; n = 55) of Finnish origin. The criminal histories of the delinquents were reviewed, and, in line with Fazel and Grann [33], murder, attempted murder, manslaughter, attempted manslaughter, arson, aggravated assault, assault, robbery, kidnapping, rape, attempted rape, and child molestation were all regarded as violent offences; and theft, burglary, driving while intoxicated, dangerous driving, driving without a driving license, as well as drug smuggling and selling drugs were regarded as non-violent offences. If the person had committed more than one offence, we took the most severe one. Of all delinquents, 83.5% (n = 506) had offended violently and 16.5% (n = 100) non-violently. Further, 42.7% (n = 259) of the delinquents had committed their index offence as minors. According to the principal psychiatric diagnoses given in the forensic psychiatric examination, 1.3% (n = 8) of the delinquents were mentally disabled (IQ < 70), 9.4% (n = 57) were diagnosed with a schizophrenia-related disorder, and 79.2% (n = 479) had a conduct disorder or a personality disorder (mainly antisocial, borderline or mixed). Further, 7.6% (n = 46) of the delinquents had some other psychiatric disorder (mainly depressive and anxiety disorders) and 2.6% (n = 16) of them showed no psychiatric disorder. Of the 606 delinquents, 45.4% (n = 275) were diagnosed with comorbid alcohol abuse or dependence.

### Controls

For each delinquent, four controls were randomly selected from the Central Population Register and matched for age, gender and place of birth. There were no exclusion criteria for this general population data.

### Follow-up

The follow-up period started when the delinquent’s forensic psychiatric examination was complete (=the index day). The delinquents and their controls were followed until 31.12.2015. The median follow-up time was 23.9 years (interquartile range = IQR 15.3–29.5).

### Mortality

The personal identification number that is assigned to all residents of Finland by the Central Population Register was used to link the data with Statistics Finland’s cause of death register. Death certificates contain information on the primary cause of death, which is always reported, and in some cases information on secondary causes. The class of death is reported as follows: attributable to a disease, an occupational disease, an accident, medical care, suicide, homicide, war, or a reason that remains unclear. In this study, deaths attributable to a disease or an occupational disease were considered natural, and those attributable to an accident, suicide or homicide were considered unnatural. It was assumed that no deaths attributable to a war would be reported in this sample, given that the Finnish–Soviet Continuation War, which was the last war in which Finland was engaged, ended in the autumn of 1944. All death certificates are verified by a physician at the regional level, and by medical experts at Statistics Finland. The register includes the deaths of all citizens and permanent residents of Finland, as well as those of Finnish citizens abroad. During the follow-up period, 53 people (15 delinquents and 38 controls) moved abroad—most commonly to other Nordic countries. They were included in the analysis on the assumption that the Statistics Finland is informed of deaths that occur abroad.

### Statistics

We used the likelihood ratio Chi square-test to compare the delinquents and their gender-, age- and place of birth-matched controls. Survival was analyzed by means of Kaplan–Meier survival plots, and group comparisons were made using the log rank test: Findings were considered significant when two-tailed p < 0.05. The odds ratio (OR) and the hazard ratio (HR) with 95% CIs were used as measures of the effect size. R version 3.3.1 (http://www.R-project.org) was used for the statistical analyses.

## Results

**Fig. 1:**
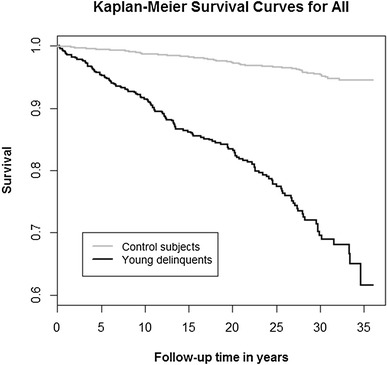
The cumulative survival of 606 delinquents and 2424 control subjects during the 36-year follow-up period

**Fig. 2:**
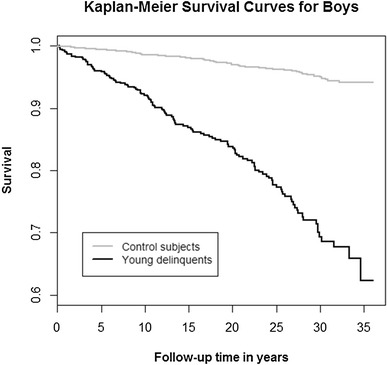
The cumulative survival of 551 male delinquents and 2204 control subjects during the 36-year follow-up period

**Fig. 3:**
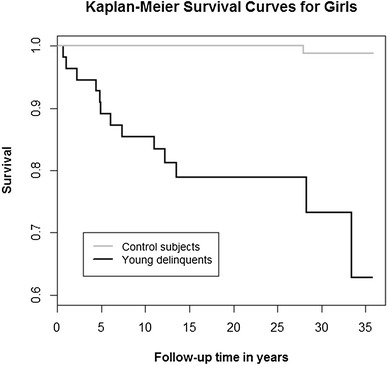
The cumulative survival of 55 female delinquents and 220 control subjects during the 36-year follow-up period

Table 1 presents the mortality of delinquents and their age-, gender-, and place of birth-matched controls. With regard to ORs, delinquents’ risk of all-cause mortality was more than eight times that of their controls. Male delinquents’ risk of all-cause mortality was more than seven times that of male controls. Among girls, delinquents’ risk of all-cause mortality was more than 67 times that of their controls. Figure 1 presents the cumulative survival of delinquents and their controls during the 36-year follow-up period. Figure 2 focuses on the cumulative survival of boys, and Fig. 3 on that of girls. With regard to different classes of death, male delinquents’ risk of unnatural death was almost 11-fold, risk of natural death more than twofold, and risk of unclear death more than fourfold compared to that of male controls. Among girls, there were no natural or unclear deaths, but the delinquents’ risk of unnatural death was more than 67-fold compared to that of their controls.

**Table 1:** Mortality of delinquents and that of their age-, gender-, and place of birth-matched controls during the follow-up

	Delinquents	Controls	OR	CI 95%	p
	n = 606	n = 2424			
All					
Deaths total; n (%)	134 (22.1)	82 (3.4)	8.11	6.05–10.86	<0.001
Natural	23 (3.8)	36 (1.5)	2.62	1.54–4.45	<0.001
Unnatural	107 (17.6)	42 (1.7)	12.16	8.40–17.60	<0.001
Unclear	4 (0.7)	4 (0.2)	4.02	1.00–16.12	0.03

	n = 551	n = 2204			
Boys					
Deaths total	121 (22.0)	81 (3.7)	7.38	5.46–9.95	<0.001
Natural	23 (4.2)	36 (1.6)	2.62	1.54–4.46	<0.001
Unnatural	94 (17.1)	41 (1.9)	10.85	7.42–15.87	<0.001
Unclear	4 (0.7)	4 (0.2)	4.02	1.00–16.13	0.03

	n = 55	n = 220			
Girls					
Deaths total	13 (23.6)	1 (0.5)	67.79	8.63–532.00	<0.001
Natural	0 (0.0)	0 (0.0)	–	–	–
Unnatural	13 (23.6)	1 (0.5)	67.79	8.63–532.00	<0.001
Unclear	0 (0.0)	0 (0.0)	–	–	–

*OR* odds ratio, *CI* confidence interval, *Natural deaths* deaths attributable to a disease or an occupational disease, *Unnatural deaths* accidents, suicides, homicides

Of the 94 unnatural deaths among the male delinquents, 45.7% (n = 43) were classified as suicides, 20.2% (n = 19) as homicides, and 34.0% (n = 32) as consequences of various accidents. Among the female delinquents, 69.2% (n = 9) of their 13 deaths were attributable to suicide, 15.4% (n = 2) to homicide, and 15.4% (n = 2) to an accident. The causes of the 36 natural deaths among delinquent males included cirrhosis of the liver, gastric bleeding, pneumonia, cardiac disease, cerebral hemorrhage, and malignancies.

Table 2 presents the comparisons between the different subgroups of delinquents. The differences between the risk of premature death among delinquents with alcohol use disorders and those without these disorders, among delinquents with schizophrenia spectrum disorders and those with conduct/personality disorders, among under-aged and young adult offenders, or among boys and girls were not statistically significant. However, when violent delinquents were compared to non-violent ones, their risk of premature death was twofold.

**Table 2:** Comparisons between different subgroups of 606 delinquents

	Deaths n (%)	HR	CI 95%	p
Gender				
Boys	121/551 (22.0)	0.85	0.48–1.51	NS
Girls	13/55 (23.6)	Ref		
Age				
Minors	50/259 (19.3)	0.76	0.54–1.08	NS
Young adults	84/347 (24.2)	Ref		
Criminal history				
Violent	121/506 (23.9)	2.01	1.14–3.57	0.009
Non-violent	13/100 (13.0)	Ref		
Diagnosis				
Schizophrenia spectrum disorders	8/57 (14.0)	0.62	0.31–1.28	NS
CD/PD	116/479 (24.2)	Ref		
Comorbid alcohol abuse/dependence				
Yes	59/275 (21.5)	1.10	0.78–1.55	NS
No	75/331 (22.7)	Ref		

*HR* hazard ratio, *CI* confidence interval, *CD* conduct disorder, *PD* personality disorder

## Discussion

Our sample appears to be highly distinct from those presented in earlier studies [8–10]: <3% of delinquents were not given a psychiatric diagnosis in the forensic psychiatric examination, and psychotic disorders were approximately three times as common as in a large meta-analysis by Fazel et al. [10]. Nevertheless, as in previous delinquency studies [8, 9], conduct and personality disorders turned out to be the most common psychiatric diagnoses. In line with the results of earlier mortality studies [1, 4], our delinquents faced a high mortality risk, and the incidence of unnatural causes of death was pronounced. The risk of premature death was comparable to that reported by Coffey et al. [1] and by Zlodre and Fazel [2], but was even higher among female delinquents. The number of female delinquents was small (<10% of the total sample), but all deaths among girls were attributed to unnatural causes.

Delinquents with a history of violent offending faced a twofold risk of premature death compared to delinquents with no such history. This finding is in line with the results of a previous follow-up study by Trumbetta et al. [7] among 14- to 15-year-old ninth graders, which indicated that the severity of delinquency is related to the risk of premature death. Moreover, a follow-up study among almost 50,000 young men recruited from the military service found that violent offending is associated with excess mortality and the risk of dying from an alcohol or drug-related cause or suicide [34]. The highest proportion of cases of death was observed among men who had committed at least one violent crime but were also victims of violence themselves [34]. In temperament studies, violently behaving offenders have scored high on novelty-seeking, impulsiveness and irritability [35]. These traits make them prone to accidents, and their risk of becoming victims of violence is high, which both lead to an increased mortality risk [36].

Schizophrenia [37], conduct disorder [15, 16] as well as antisocial [38] and borderline [39] personality disorders are all associated with excess mortality. Contrary to our hypothesis, we were unable to find a substantial difference between the risk of premature death among delinquents diagnosed with schizophrenia spectrum disorders and among those diagnosed with conduct or personality disorders. This finding is interesting, given that the Finnish correction system singles out those who suffer from a psychotic disorder and refers them to psychiatric hospitals, where the treatment periods frequently last for several years. This was also the case among our delinquents with schizophrenia spectrum disorders: they were all evaluated as lacking criminal responsibility and sent for involuntary psychiatric inpatient treatment. This procedure did not seem to help in terms of reducing their premature mortality, however, when they were compared to antisocially behaving delinquents who were not similarly treated. Indeed, low socioeconomic status, unfavorable health behaviors such as smoking and drug and alcohol abuse, as well as self-destructive behaviors and suicide tendencies are all linked to offender populations in general, regardless of individual psychiatric diagnoses. Some authors have identified antipsychotic medication as a cause of excess mortality [40], but others have reported the opposite finding [41].

Antisocial personality disorder is linked to Cloninger’s type 2 alcoholism, which is characterized by strong physical dependence that typically already develops in adolescence [21]. Almost half of our delinquents were diagnosed with alcohol abuse or dependence, which most probably mirrors this well-known relationship. Contrary to our hypothesis, we observed no significant difference between the all-cause mortality of those who were diagnosed with alcohol use disorders and those who were not. Unfortunately, we had no information on offenders’ later alcohol use, but we assume that many of those with no alcohol use disorder at the time of the forensic psychiatric examination might have been diagnosed accordingly later in life, which might explain our finding.

The majority of Finnish under-aged delinquents with severe offending are referred to child-welfare institutions such as residential schools, which focus on rehabilitation rather than punishment. Education, for example, has a high priority in these institutions. The residential school placement ends at the age of 18, when the adolescents are offered a voluntary 5-year after-care program [42]. Contrary to our hypothesis, the risk of premature death did not significantly differ between delinquents who had committed their index crimes under-aged and those who had committed them as young adults (aged 18 or 19). However, many of our young adult offenders had also been clients of child-welfare during their childhood or adolescence; hence we cannot draw any final conclusions regarding the role of child-welfare in reducing later mortality risk. However, Manninen et al. [43] recently reported that Finnish adolescents placed in residential schools show a substantially elevated mortality risk in young adulthood compared to that of the general population. The authors concluded that to reduce mortality, more effective treatment of mental and substance-use-related problems during and after the residential school placement is needed.

Contrary to our hypothesis, there was no substantial gender difference between the all-cause mortality of male and female delinquents. We have previously shown that when male and female delinquents matched for age and type of crime are compared to each other, the females resemble their male counterparts in many psychosocial adversities [44]. The present result adds to this with regard to mortality.

### Strengths and limitations

The main strength of this study lies in its nationwide and comprehensive nature. The Finnish tradition of thorough forensic psychiatric examination and reliable statistics constitute a solid basis for register-based studies. The clearance rate for violent crimes in Finland is high [45]. The proportion of females was low, but corresponds quite well to the proportion of females who commit violent crimes, as reported by national statistics on police-investigated crime and self-reported juvenile delinquency [46]. The estimate of female delinquents’ mortality risk must be regarded as unreliable, as there was only one death among the female controls. In addition, some 95% CIs were quite large. The median follow-up period was substantial, almost 24 years. As in many register-based follow-up studies, the controls were matched according to age, gender and place of birth, but not for socio-economic status or trauma history, both of which are known to increase mortality risk [47–49]. It should also be borne in mind that the courts decide who is sent for a pretrial forensic psychiatric examination, and that these decisions could be described as somewhat arbitrary. We know that the majority of homicidal adolescents undergo this examination [50]. With regard to other crime types, an adolescent is typically sent for an examination, if the court expects that he/she might be suffering from a mental health disorder, but this decision is often decided without the benefit of medical expertise. Even though we collected delinquents’ primary psychiatric diagnoses as well as alcohol use disorder diagnoses, we did not assess the severity of psychopathological symptoms. Our sample comprised only 16 delinquents with no psychiatric disorder; thus, we did not compare psychiatric and non-psychiatric delinquents. To shed light on this issue, new studies with sufficiently large sample sizes are needed. All the pretrial offenders were Finns by origin, thus the results cannot be generalized to immigrants.

## Conclusions

Even though the Finnish correction system prefers psychiatric treatment and rehabilitation over criminal sanctions, and the national health care system offers developmental-phase-specific psychiatric care, the mortality rate of delinquents, especially of those with a history of violent offending, is high. The excess mortality of offenders can be regarded as a specific public-health inequity that calls for more effective intervention procedures than those used thus far.

## Abbreviations

CI: confidence interval
DSM: the Diagnostic and Statistical Manual of Mental Disorders
HR: hazard ratio
ICD: International Classification of Diseases
OR: odds ratio
